# Metagenomic investigation of bacteria associated with dental lesions: a cross-sectional study

**DOI:** 10.4317/medoral.23326

**Published:** 2020-02-10

**Authors:** Ali Kazemtabrizi, Azam Haddadi, Mahmoud Shavandi, Naser Harzandi

**Affiliations:** 1 Ph.D candidate, Department of Microbiology, Karaj Branch, Islamic Azad University, Karaj, Iran; 2 Assistant Professor, Department of Microbiology, Karaj Branch, Islamic Azad University, Karaj, Iran; 3 Assistant Professor, Environment and Biotechnology Research Division, Research Institute of Petroleum Industry, Tehran, Iran; 4 Assistant Professor, Department of Microbiology, Karaj Branch, Islamic Azad University, Karaj, Iran

## Abstract

**Background:**

Dental caries is considered as one of the most significant global health problem over the world. Dental caries initiates from bacterial shifts within the supragingival biofilm, then a polymicrobial biofilm is formed on the surface of tooth, and finally various bacterial species aggregate in a complex-organized manner. The exploiting variability in 16S rRNA gene sequence has been considered as a cost-efficient high-throughput characterization approach in human oral microbiome investigations. The aim of this study is to characterize bacterial species associated with superficial dental biofilm, underlying carious dentine and root caries lesion by16S rRNA gene-based metagenomic analysis.

**Material and Methods:**

Herein, the bacterial communities in carious dentin lesion, biofilm and root canal samples of 30 subjects (aged 4–76 years) admitted to a clinic in Tehran during 2017 were investigated using a culture independent approach. Total genomic DNA of each tissue was subjected to metagenomic identification of bacteria using a nested PCR assay and 16S rRNA library construction method.

**Results:**

31 samples collected from 30 consenting patients (29 samples from 29 patients ant two biofilm samples from one patient). Bioinformatics analyses of a-800bp sequences of the second step of Nested-PCR revealed presence of 156 bacterial isolates in carious (n=45), biofilm (n=81) and root canal (n=30) specimens. *Prevotella* spp., *Lactobacillus vaginalis*, and *streptococcus* spp. showed higher prevalence in carious dentin, root and biofilm samples, respectively.

**Conclusions:**

Exploring the dental microbiota and comparing them in health or diseased conditions is critical step in the determination of human general health. The method applied in this study could identify bacteria related to the three dental lesions. However, due to lack of data for comparison in Genbank or because of the sequence similarity lower than 98% for most identified bacteria, the use of more powerful approaches like NGS platforms or typing of multiple loci (MLST) in future studies is recommended.

** Key words:**Bacterial composition, dental caries, dental biofilm, oral microbiome.

## Introduction

The oral cavity is considered as one of the most complex microbial environments, harboring hundreds of bacterial species that play important role in maintaining oral homeostasis and developing various oral diseases, particularly dental caries and periodontal disease (1). Dental caries is considered as one of the most significant global health problem that affects people of all age groups over the world (2). This disorder is a multifactorial disease resulting from interactions between a susceptible host, cariogenic microorganisms, and cariogenic diets. Dental caries initiates from bacterial shifts within the supragingival biofilm, then a polymicrobial biofilm is formed on the surface of tooth, and finally results in aggregation of various bacterial species in a complex-organized manner (3).

The primary oral pathogens associated with dental caries in humans are **Streptococcus* mutans* and *S. sobrinus*, which considered the main cariogenic pathogens for decades. Other dental caries-associated pathogens include non-mutans streptococci, *Actinomyces*, *Lactobacillus*, *Veillonella*, and *Bifidobacterium spp*. (4). Now, it is extensively accepted that a shift or dysbiosis in oral bacterial communities is a major risk factor for dental caries (5).

Notwithstanding its considerable impact on human health, the diversity and composition of the oral bacterial population and its changes during shifting between the healthy and diseased states are far from being fully understood (6). Moreover, oral microorganisms may be important pathogens playing role in systemic diseases, including cardiovascular disease, gastrointestinal and colo-rectal cancer, respiratory tract infection, diabetes, and adverse pregnancy outcomes (7). The association between periodontal disease and severe systemic disease may be due to both translocation of bacteria into the bloodstream and increased systemic inflammation (8).

Although conventional culture dependent techniques have been used to isolate and identify about 300 oral bacterial species, the majority of oral microbiome cannot be cultivated *in vitro* (9). Recently, molecular approaches are extensively used to investigate the oral microbial community structure, including the identification and characterization of culturable and non-culturable bacteria with higher resolution than was previously possible with culture-based techniques (10). Metagenomics using microbial 16S ribosomal RNA (rRNA) gene sequencing has produced bacterial profiles and genomic profiles to study the relationships between microbial diversity and oral diseases (11). The exploiting variability in 16S rRNA gene sequence has been considered as a cost-efficient high-throughput characterization approach in human oral microbiome investigations (12). The 16S rRNA gene is uniquely found in bacteria and considered as a barcode that can be used to detect individual bacterial species, identifying the broad spectrum of both culturable and non-culturable bacteria (13).

Recently, it was found that oral microbiota are different in various geographic regions (14) and ethnicities (15). In addition, as far as we know, nobody has been published study about oral microbiome related to dental caries and periodontal disease by metagenomic analysis in Iran. Since the oral microbiota diversity is considered as the main etiologic factor for dental caries developing, several further studies should be conducted to better understand this dental problem. Thus, the aim of this study, was to identify bacteria involved in superficial dental biofilm, underlying carious dentine and root caries lesion in adult by16S rRNA gene-based metagenomic analysis and increased information about this important subject.

## Material and Methods

- Patients and samples

The present study was conducted on the biofilm (n=14), caries dentin (n=12) and root canal (n=5) samples which collected from 30 consenting patients (29 samples from 29 patients and two biofilm samples from one patient) who admitted to the Dental Clinic in East Tehran from February to December 2017. The prepared check list was obtained from each patient carefully. Accordingly, in all cases, demographical information such as age, sex, and underlying clinical conditions were recorded (Table 1). Biofilm samples were taken from patients who only had been examined by the dentist at the clinic visiting, decay samples from patients who had dental restorations, and root canal samples, from patients who had endodontic therapy. A carious tooth was isolated with a rubber dam from each subject. After removing dental biofilm constituents, enamel and a narrow layer of dentin were collected with a sterile diamond bur. Specimens from large carious dentin were grouped according to depth, placed into sterile vials containing phosphate-buffered saline (PBS), and analysed individually. The root canal sampling was performed using exploiting sterile burs under manual irrigation with sterile PBS. A sterile paper point was implanted to collect the specimen from the pulp chamber following the root canal preparation under aseptic conditions. The paper points were maintained in sterile PBS. All of the samples were stored at –70 oC for further analysis.

**Table 1:**
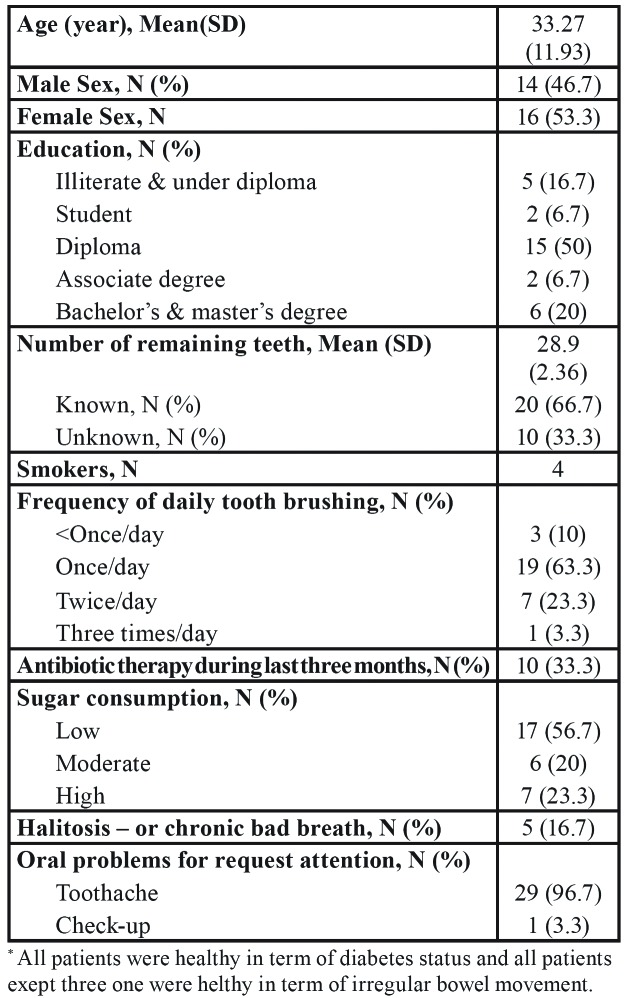
Demographic and clinical characteristics of included persons in this study.

- 16S rRNA gene amplification

Total DNA was extracted from each sample using MBST DNA extraction kits (Investigating Unit Molecular Biological System Transfer, Tehran, Iran) according to the manufacturer’s instructions. Evaluation of concentration and purity of the extracted DNA was performed by ND-1000 spectrophotometer (Thermo Scientific, Waltham, MA, USA) and agarose gel electrophoresis. Finally, eluted DNA was stored at -20°C for PCR analysis. The 16S rRNA gene amplification was carried out by a nested PCR approach. Briefly, first, a 1500 bp fragment of 16S rRNA gene was amplified using the Nest1 primer pair as followed: Nest1 F: GAGTTTGATCCTGGCTCAG; and Nest1 R: GTTACCTTGTTACGACTT. The PCR reactions were performed in a total volume of 25 μl containing 12.5 μl of the master mix, 1μl of each primer, 2μl of DNA template and 8.5 μl of distilled water. The amplification conditions were initial denaturation at 94°C (10 min) and 35 cycles consisting of 95°C (30 sec), 57.5 °C (40 sec), 72°C (30 sec) and a final extension at 72°C (8 min). Another primer pair set that used for amplyfication of 800 bp fragments of the 16S rRNA gene were Nest2 F: GCRKGCCTAAYACATGCAAG; and Nest2 R: CGTGGACTACCAGGGTATCT. The program for this amplification was 94°C (5min), 10 cycles consisting of 94°C (30 sec), 58°C (30 sec), 72°C (90 sec), 25 cycles consisting of 94°C (30 sec), 57°C (30 sec), 72°C (90 sec) and an additional extension time at 72°C (10 min).

PCR products of the Nest2 primer pair were analyzed by electrophoresis on 1% agarose gel, Gelred staining and evaluated by UV transilluminator (Gel doc system UVItec, Cambridge, UK). Finally, the PCR band related to 800bp fragments were purified by GeneAll PCR purification kit (GeneAll Biotechnology Co, Korea).

- Cloning and sequencing

The purified 800bp amplicons were TA-cloned into the pTG19-T cloning vector. The ligation reaction of the amplicons was done into the vector according to the manufacturer’s instructions (Fermentas, Lithuania). After ligation, the DNA construct mixture was transformed into the electrocompetent *E. coli* DH5a cells and cultured in the LB plate with the suiTable concentration of ampicillin. Finaly, the transformants were checked by colony PCR analysis using gene specific primers and the *BamHI* restriction enzyme analysis. Positive insert clones were selected and after plasmid extraction by GeneAll plasmid extraction kit (GeneAll Biotechnology Co, Korea), analyzed by electrophoresis on 0.8% agarose gel.

After electrophoresis and ensuring the presence of plasmid, the extracted plasmid was sequenced by the Sanger's method using the M13 forward and reverse pTG19-T specific sequencing primers. For each sequence data, the nearest-neighbor species with >98% identity were first searched via the Basic Local Alignment Search Tool (BLAST) using blastn function in National Center for Biotechnology Information (NCBI) with the default parameters in megablast comparing with the reference sequences in 16S rRNA RefSeq (http://www.ncbi.nlm.nih.gov/BLAST/). Furthermore, the sequences with no hits were compared with the Nucleotide database (nr/nt) of the NCBI GenBank using blastn optimized for megablast with default parameters.

## Results

To characterize the bacterial population diversity in the obtained lesion samples of patients with dental caries, we checked the total genomic DNA extracted from 31 lesion samples of 30 patients with polycaries lesions. In this study, the most microbial load belonged to biofilm samples (81 colonies) and the lowest belonged to root samples (30 colonies). A total of 260 isolates were recovered and after examination similar sequences were removed. Subsequently, 156 isolates were confirmed.

Accordingly, 45 colonies were obtained from carious dentin samples with the majority of them (24.4%) belonging to *Prevotella* spp. Additionally, *Shuttleworthia*, *Streptococcus*, and *Campylobacter* were present in much lower proportions. The 16S rRNA sequence analysis revealed 11 new bacterial families, 4 new genera and 29 species detected from carious dentin samples (Table 2). The evaluation of 5 root samples indicated that a majority of the recovered colonies (55.3%) belonged to new bacterial strains (defined as strains sharing over 98% 16S rRNA gene sequence identity), including 15 new families, one new genus and 13 new species, which are closely related to **Olsenella* profuse*, followed by *Lactobacillus* vaginalis (40%) (Table 3). In addition, our analysis demonstrated that 54% of bacterial isolates from biofilm samples belonged to new bacterial strains, including 20 new families, 17 new genera and 44 new species (Table 4).

**Table 2:**
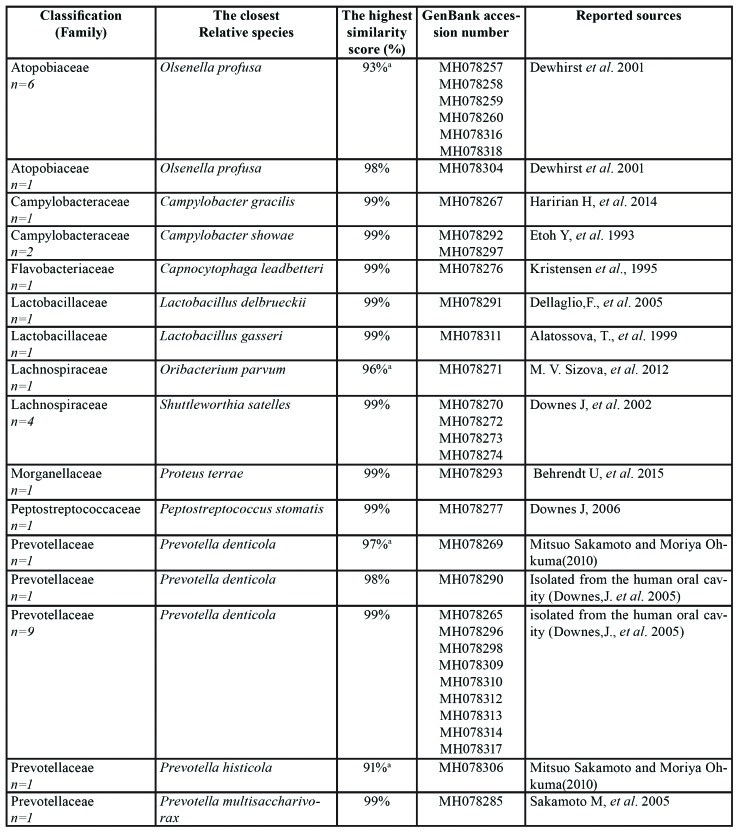
Bacterial strains identified in 12 carious dentin samples (n=45).

**Table 2 cont.**
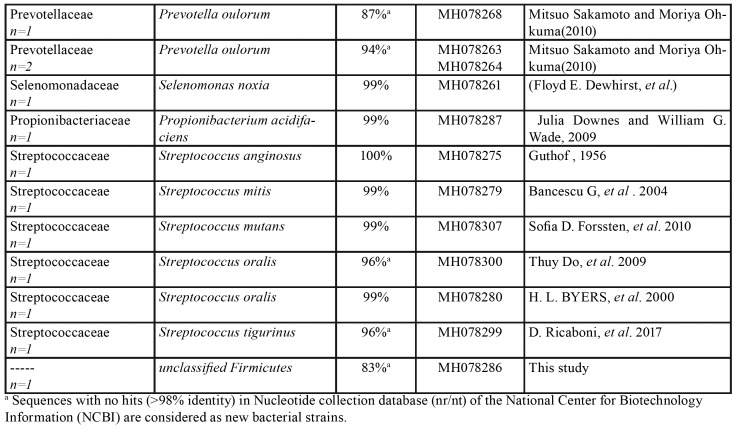
Bacterial strains identified in 12 carious dentin samples (n=45).

**Table 3:**
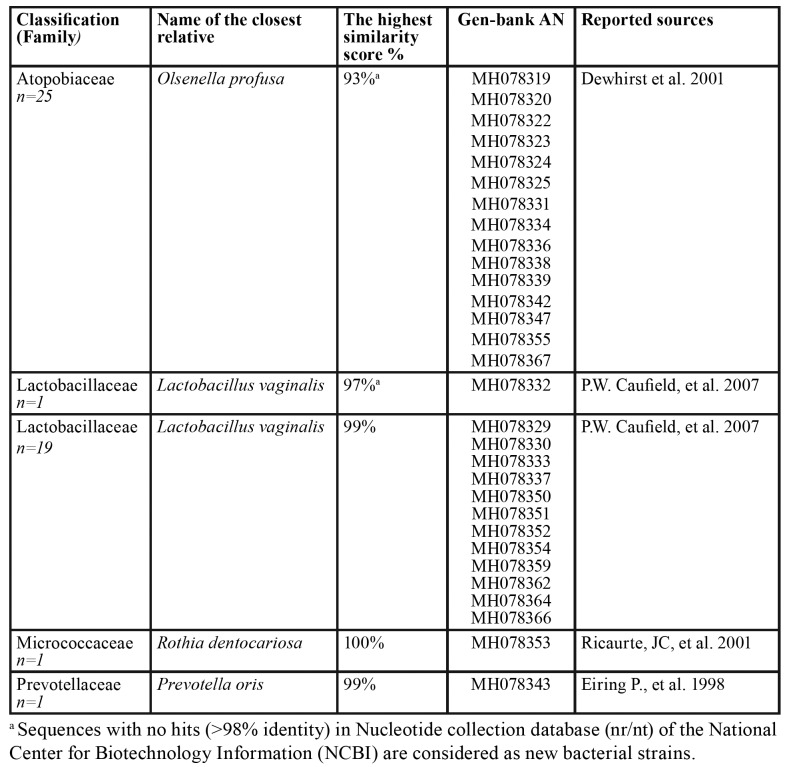
Bacterial strains identified in 5 root samples samples (n=30).

**Table 4:**
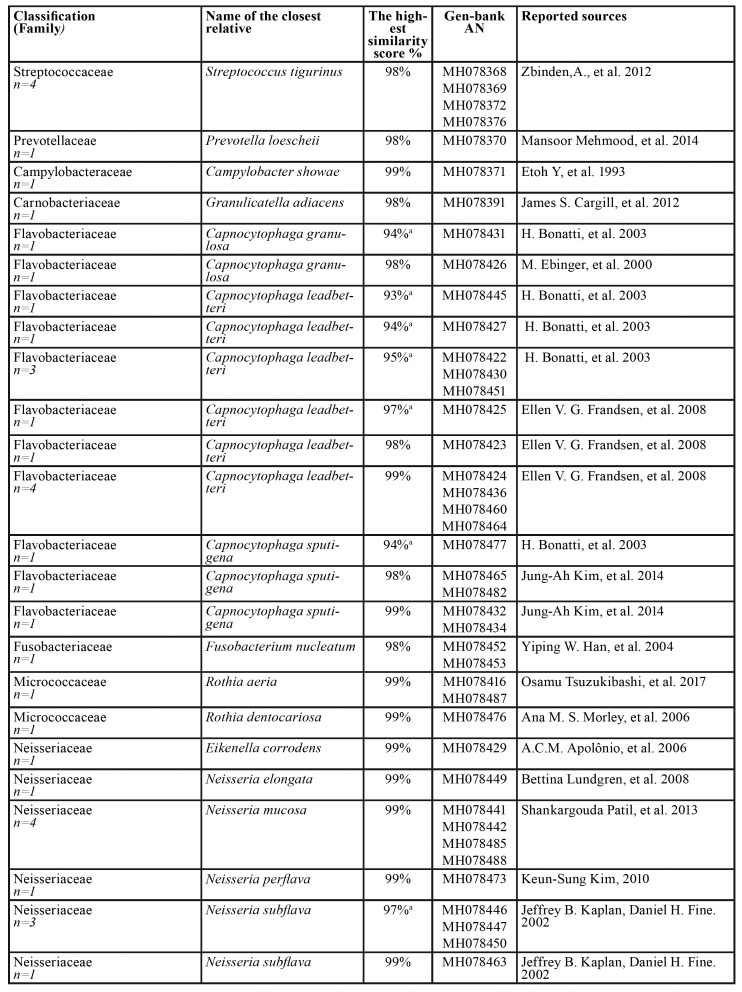
Bacterial strains identified in 14 biofilm samples (n=81).

**Table 4 cont.**
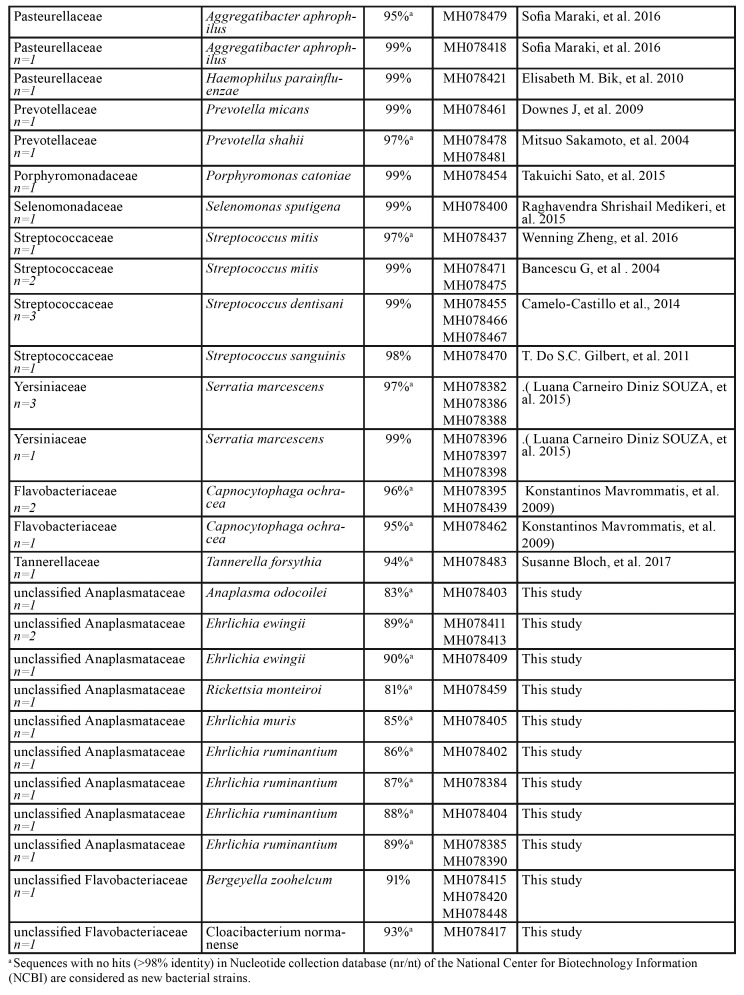
Bacterial strains identified in 14 biofilm samples (n=81).

**Figure 1:**
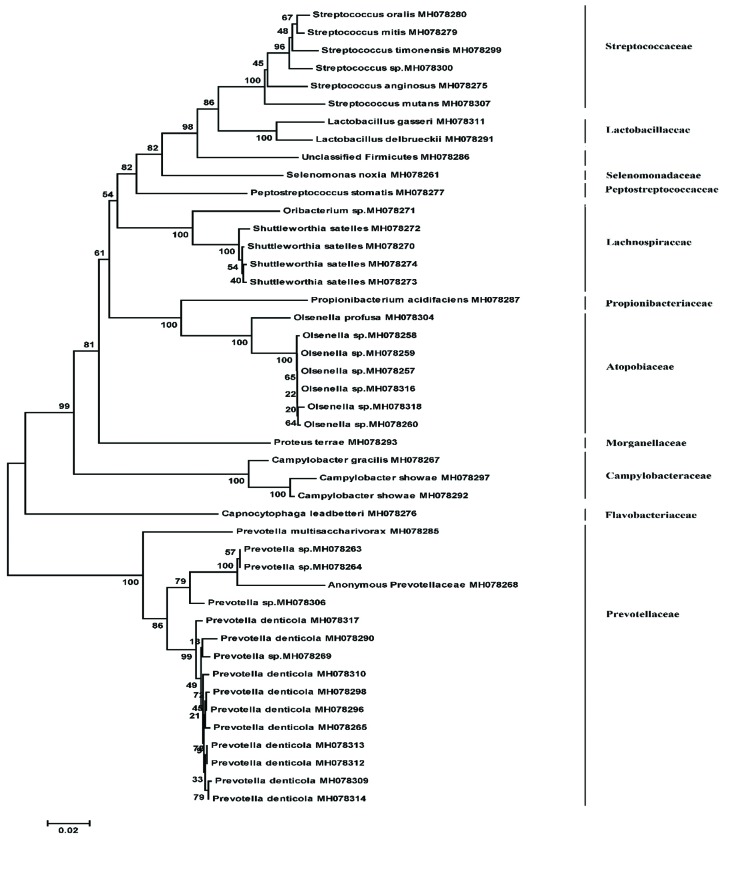
Phylogenetic relationship among 16S rRNA gene sequences retrieved from carious dentin samples by neighbour joining method. The numbers given at nodal points indicate bootstrap values (as percentages) for 1000 replications.

**Figure 2:**
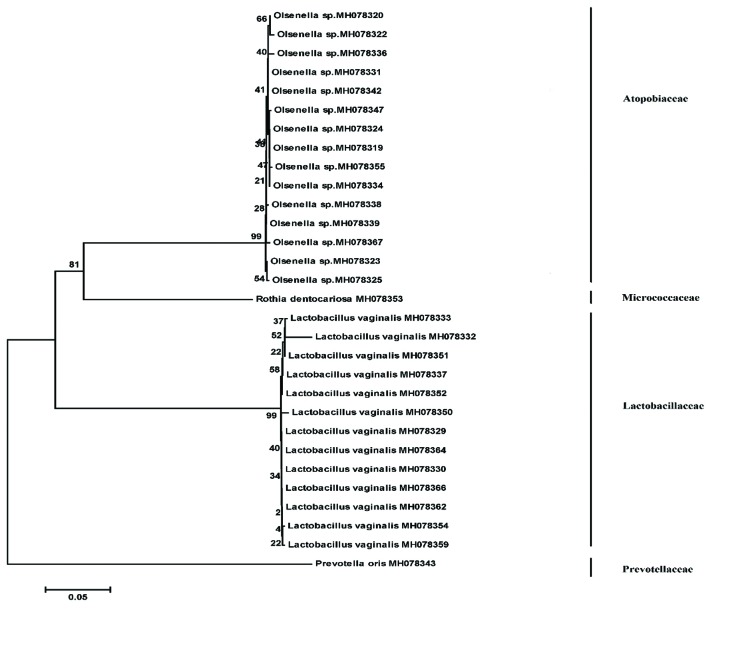
Phylogenetic relationship among 16S rRNA gene sequences retrieved from root samples by neighbour joining method. The numbers given at nodal points indicate bootstrap values (as percentages) for 1000 replications.

**Figure 3:**
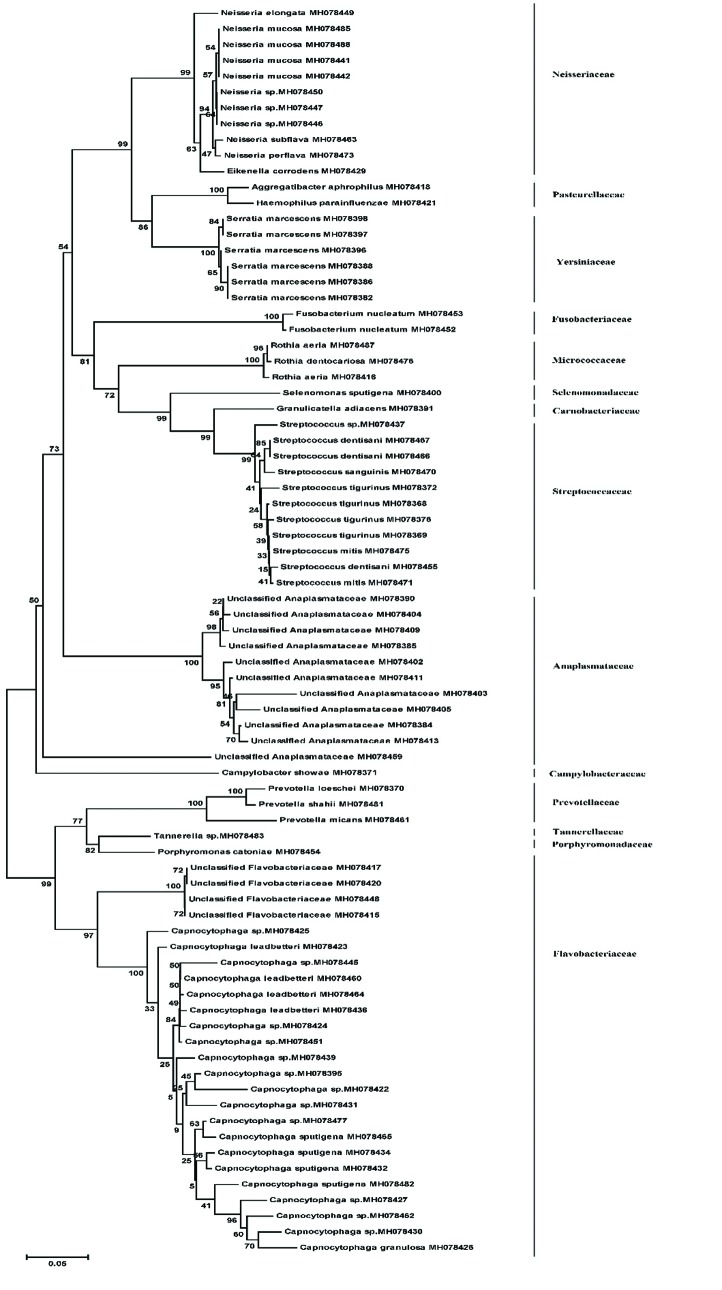
Phylogenetic relationship among 16S rRNA gene sequences retrieved from biofilm samples by neighbour joining method. The numbers given at nodal points indicate bootstrap values (as percentages) for 1000 replications.

The phylogenetic relationship among bacterial strains isolated from decay, root and biofilm samples are shown in Fig. 1, Fig. 2, and Fig. 3, respectively.

## Discussion

With the development of various molecular approaches, bacterial diversity and microbiota composition in different microenvironments have been explored using genome analysis such as sequence analysis of microbial 16S rRNA genes and other universal targets (e.g., cpn60) (16,17). Improvements in sequencing-based techniques allow a dramatic increase in the quality of analysis of large scale samples with limited sample processing and lower costs in comparison to conventional methods (18). In the present study, we investigated the composition of the microbiota composition in dental biofilm, carious dentine lesions and root samples using PCR amplification of bacterial 16S rRNA gene followed by sequencing. Our main finding was the differences in bacterial species presented in biofilm, carious dentine and root samples obtained from different patients. Indeed, the differences in co-occurrence patterns of taxa between biofilm, carious dentine lesions and root canal samples support a more complex etiology of disease than a simple progression in dental caries. In our study, distinct bacterial communities were detected when comparing the bacterial profile of biofilm, carious dentine and root samples.

A comprehensive and thorough understanding of the microbial diversity of biofilms is crucial for developing effective prevention and treatment strategies of dental caries (19). The proportion of the variability in the microbial population of dental biofilm within and across various geography is often uncharacterized. In the current study, a majority of bacterial strains detected in biofilm samples from studied patients classified as new bacterial species, followed by streptococci and *Capnocytophaga* spp. It is now documented that there are a large variety of microbial species obviously occurring in the oral biofilm, which produce acid from different carbohydrate substrates (20). Several studies have established that pH is an imperative factor affecting the microbial composition of biofilm and that the production of various organic acids from carbohydrate fermentation by lactic acid bacteria (such as mutant streptococci and *lactobacilli*) and associated reduction in pH can inhibit the growth of surrounding microorganisms including non-pathogenic bacteria (21). Although, the data obtained from analysis of dental biofilms by Peterson *et al*., in which they demonstrated that this ecological niche is an extremely selective environment as they observed only four distinct phyla and a relatively small number of known genera (n=36) (22), another study have investigated various phyla and genera in biofilms in greater depth (23). Based on patients with confirmed dental caries in our study could suggest an increased risk for dental caries in persons presenting higher levels of *Campylobacter*, streptococci, *Lactobacillus* and *Prevotella*.

In this study, the families with the highest number of bacteria detected from root canal samples were *Atopobiaceae* and *Lactobacillaceae* in phylum Firmicutes. Some studies previously reported phylum Firmicutes is dominant in Europe and Asia regions (24,25). At the genus level, our analysis showed that unclassified *Olsenella* was found in relatively high abundance in carious dentin and root canal infections. The current result is not in a good agreement with many other published researches showed that *Prevotella*, *Parvimonas*, *Atopobium* and *Porphyromonas* are predominant bacterial genera found in root canal samples (26-28). Moreover, Pourhajibagher *et al*. previously reported that **Veillonella* parvula*, *Enterococcus faecalis*, and **Porphyromonas* gingivalis* were found to be predominant bacteria in endodontic infections among 50 Iranian patients with endodontic infections (29). The presence of *Lactobacillus* vaginalis in root canal samples in the present study possibly suggests that its role on the development, maintenance and relapse of periapical infections may be important. Our findings also indicated that the bacterial diversity in carious dentin, root and biofilm samples are different.

In a review article by Bui *et al*. explained that there is an association between periodentitis and systemic disease (7). A number of bacteria identified in the present study that may contribute to systemic disease are listed in the review study. These diseases include cardiovascular disease (*Eikenella corrodens*, *Tannerella forsythia*, *Fusobacterium nucleatum*), gastrointestinal and colorectal cancer (*Fusobacterium nucleatum*), diabetes and insulin resistance (none of samples), and Alzheimer's disease (*Fusobacterium nucleatum*, *Tannerella forsythia*), as well as respiratory tract infection (*Capnocytophaga*, *Eikenella corrodens*, *Fusobacterium nucleatum*) and adverse pregnancy outcomes (*Fusobacterium nucleatum*). The present study does not provide patients’ information about the comorbidities of patients, so the role of oral infections in systemic diseases cannot be investigated clearly and further studies are needed.

Conclusion

We were able to pinpoint several caries-related genera included *Campylobacter*, *Capnocytophaga*, *Lactobacillus* and *Streptococcus*. Detection of unclassified bacterial strains related to *Olsenella* genus was remarkably observed in current study. Therefore, the bacterial diversity found in the current study could be considered in the treatment strategy of caries in the study area however; a large scale study utilizing advance methods like NGS plat forms or typing of bacteria vie the multiple loci (MLST) is recommended. In addition, a comparison of this study to other published researches indicate a significant heterogeneity in study outcomes and suggest that novel approaches are necessary to further define the microbial composition of dental caries onset and progression. Although, we should point out here, that only caries sites were investigated for the present evaluation and thus, further studies should focus on microbiota composition of caries samples in comparison to those found at intact enamel sites among the Iranian population.
